# Author Correction: SARS-CoV2-mediated suppression of NRF2-signaling reveals potent antiviral and anti-inflammatory activity of 4-octyl-itaconate and dimethyl fumarate

**DOI:** 10.1038/s41467-020-19363-y

**Published:** 2020-10-21

**Authors:** David Olagnier, Ensieh Farahani, Jacob Thyrsted, Julia Blay-Cadanet, Angela Herengt, Manja Idorn, Alon Hait, Bruno Hernaez, Alice Knudsen, Marie Beck Iversen, Mirjam Schilling, Sofie E. Jørgensen, Michelle Thomsen, Line S. Reinert, Michael Lappe, Huy-Dung Hoang, Victoria H. Gilchrist, Anne Louise Hansen, Rasmus Ottosen, Camilla G. Nielsen, Charlotte Møller, Demi van der Horst, Suraj Peri, Siddharth Balachandran, Jinrong Huang, Martin Jakobsen, Esben B. Svenningsen, Thomas B. Poulsen, Lydia Bartsch, Anne L. Thielke, Yonglun Luo, Tommy Alain, Jan Rehwinkel, Antonio Alcamí, John Hiscott, Trine H. Mogensen, Søren R. Paludan, Christian K. Holm

**Affiliations:** 1 Department of Biomedicine, Aarhus Research Center for Innate Immunology, Aarhus University, Aarhus, Denmark; 2 Department of Infectious Diseases, Aarhus University Hospital, Aarhus, Denmark; 3 Centro de Biología Molecular Severo Ochoa (Consejo Superior de Investigaciones Científicas - Universidad Autónoma de Madrid), Nicolás Cabrera 1, 28049 Madrid, Spain; 4 Medical Research Council Human Immunology Unit, Medical Research Council Weatherall Institute of Molecular Medicine, Radcliffe Department of Medicine, University of Oxford, Oxford, OX3 9DS UK; 5 Omiics ApS, Åbogade 15, 8200 Aarhus N, Denmark; 6 Children’s Hospital of Eastern Ontario Research Institute, Department of Biochemistry Microbiology and Immunology, University of Ottawa, Ottawa, ON K1H 8L1 Canada; 7 Department of Chemistry, Aarhus University, Aarhus, Denmark; 8 Fox Chase Cancer Center, 333 Cottman Avenue, Philidelphia, PA 19111-2497 USA; 9 Lars Bolund Institute of Regenerative Medicine, BGI-Shenzhen, Shenzhen, 518083 China; 10 Department of Biology, University of Copenhagen, 2100 Copenhagen, Denmark; 11 Department of Pediatrics and Adolescent Medicine, Division of Pediatric Neurology, University Medical Center Göttingen, 37075 Göttingen, Germany; 12 Istituto Pasteur Italia-Cenci Bolognetti Foundation, Viale Regina Elena 291, 00161 Rome, Italy; 13 Department of Clinical Medicine, Aarhus University, Aarhus, Denmark

**Keywords:** Immunology, Microbiology, SARS-CoV-2

Correction to: *Nature Communications* 10.1038/s41467-020-18764-3, published online 2 October 2020.

The original version of this Article contained an error in the spelling of the author Trine H. Mogensen, which was incorrectly given as Trine Mogensen. This has now been corrected in both the PDF and HTML versions of the Article.

