# Supplementary material for: SARS-CoV2-mediated suppression of NRF2-signaling reveals potent antiviral and anti-inflammatory activity of 4-octyl-itaconate and dimethyl fumarate
Source: Nat Commun. 2020 Oct 2;11:4938. doi: 10.1038/s41467-020-18764-3 (PMC7532469; doi:10.1038/s41467-020-18764-3)
Supplement: Supplementary file 1 — Supplementary Information [file 41467_2020_18764_MOESM1_ESM.pdf]

Title: SARS-CoV2-mediated suppression of NRF2-signaling reveals potent antiviral and anti-inflammatory activity of 4-octyl-itaconate and dimethyl fumarate

Olagnier et al., 2020

This PDF file includes:

Supplementary Figures 1-11

Supplementary Figure 1.

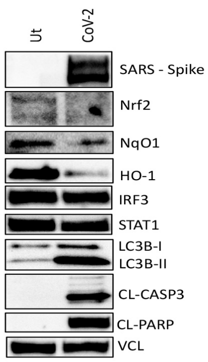

Fig. S1. NRF2 inducible genes are repressed during infection with SARS-CoV2 in Vero cells. Vero cells were either left untreated or infected with SARS-CoV2 for 24 hours before analysis of cell pellets by immunoblotting using indicated antibodies. Experiment is one representative of two independent experiments.

Supplementary Figure 2.

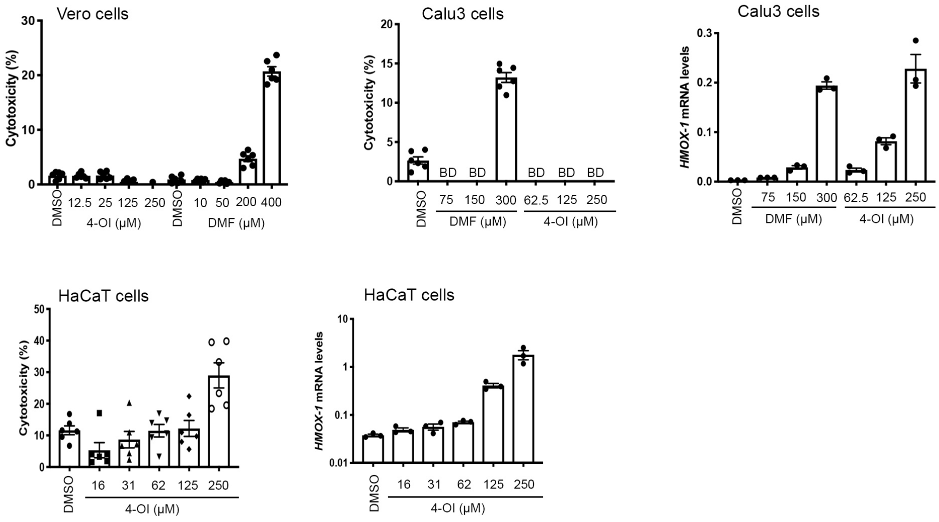

Supplementary Figure 2. Cytotoxicity of 4-OI and DMF
Indicated cell lines were treated with either 4-OI or DMF for 48 hours before analysis of cell supernatants by LDH assay and of cell pellets by qPCR for NRF2-inducible gene HO-1 (*HMOX-1*). Cytotox data on dispaly are pooled data from two independent experiments each performed in biological triplicates. Qpcr data on display are from one experiment performed in biological triplicates. All bars indicate mean and all error bars indicate s.e.m.

Supplementary Figure 3.

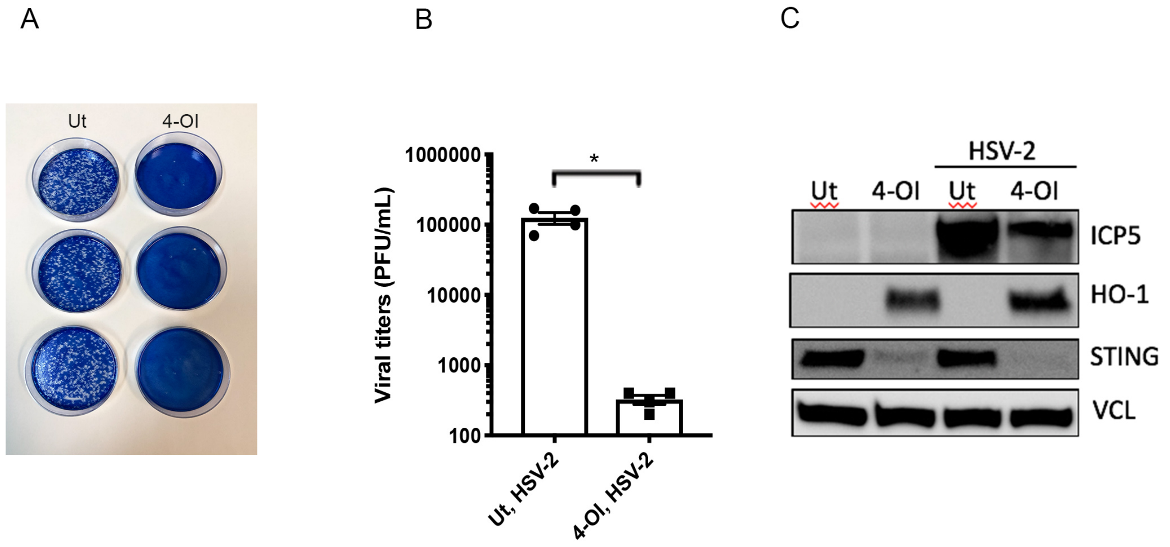

Supplementary Figure 3. 4-OI inhibits HSV2 replication
HaCaT cells were treated with 4-OI at 125µM for 48 hours before infection with HSV2 at MOI 0.01. Cell supernatants were then harvested to determine release of progeny virus by plaque assay (A+B) and cell pellets were lysed for immunoblotting using specific antibodies against HSV protein ICP5, Heme Oxygenase 1 (HO-1), STING, and Vinculin as loading control(C). A) is a photo of plaque assay performed on vero cells. B) is a quantification of the plaque assay. Graph displayed in B is pooled data from two independent experiments each performed in duplicates. Bars indicate means and error bars indicate s.e.m. Statistical analysis was performed using a two-tailed Mann-Whitney test where $p=0.00286$.

Supplementary Figure 4.

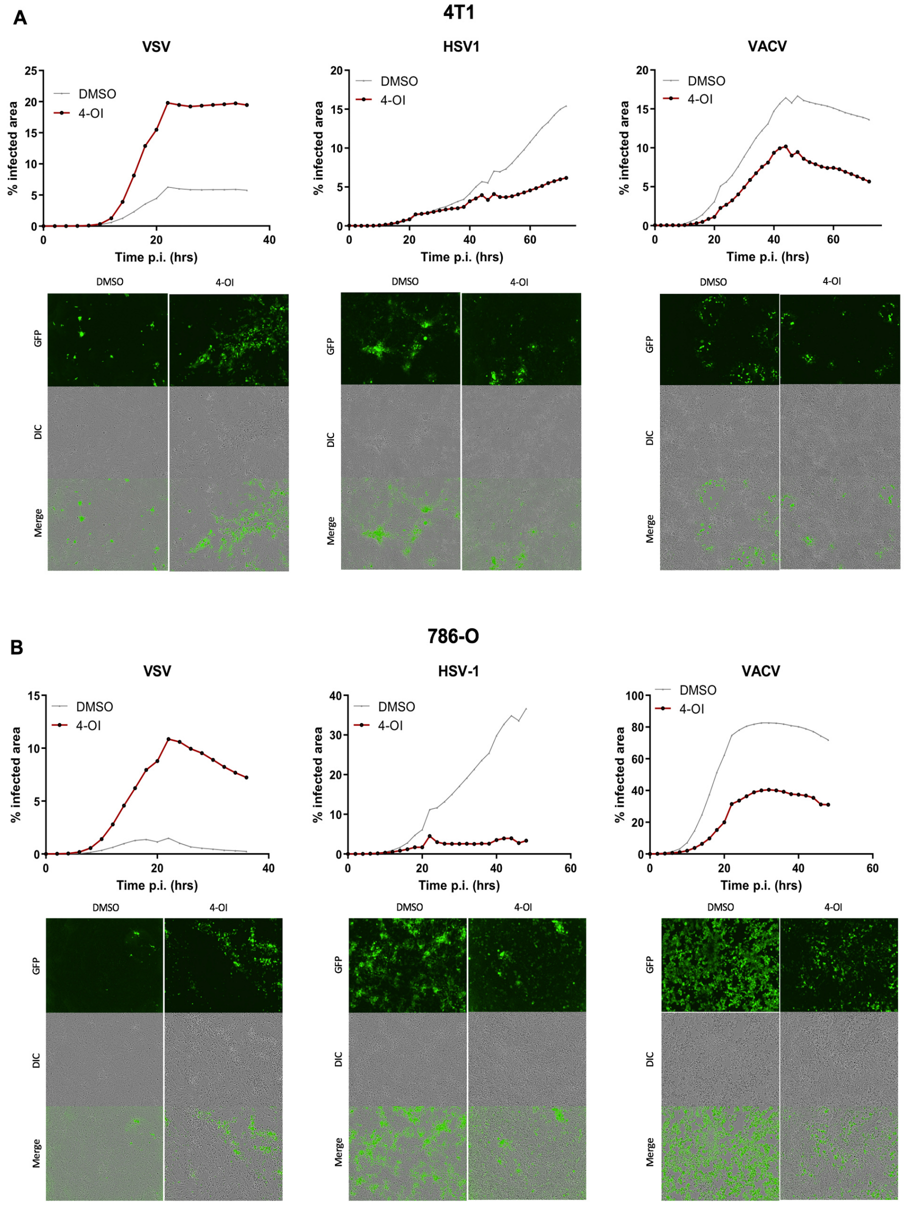

Supplementary Figure 4: 4-OI impairs HSV and VACV spreads in cancer cells but enhances VSV. (A) Mouse breast cancer cells (4T1) or (B) human renal carcinoma cells (786-O) were treated with 4-OI (150 μM) for 48 hours, then infected with VSV-Δ51-GFP, HSV-ΔICP0-GFP or VACV-GFP at MOI of 0.1. Upper line charts: Live virus spreads were monitored via GFP fluorescence signal using the Incucyte Live-Cell imaging system, images were taken every 2 hours using 10x magnification. Lower images: representative fluorescence microscopy images of virus infection at 24 hours p.i. for VSV and 48 hours p.i. for HSV-1 and VACV. Data are representative of two independent experiments.

Supplementary Figure 5.

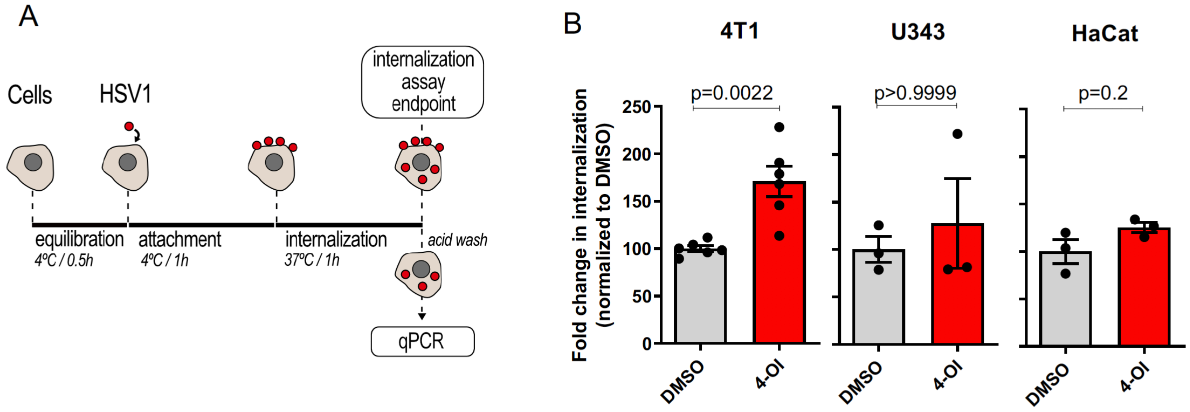

Supplementary Figure 5. 4-OI enhances HSV-1 internalization. (A) Schematic representation of the internalization assay performed in a breast cancer cell line (4T1), in human glioblastoma (U343), and in immortalized human keratinocyte (HaCaT). Cells were pretreated with 4-OI (150 μM) for 48 hours, then the viral entry assay was carried out with HSV-1 incubation at an MOI of 10. (B) Graphs of HSV-1 internalization in 4T1, U343 and HaCaT cells. Bars indicate mean with SEM from at least 3 independent experiments. Statistical analysis was performed Mann-Whitney test, p-values are reported in each graph.

Supplementary Figure 6.

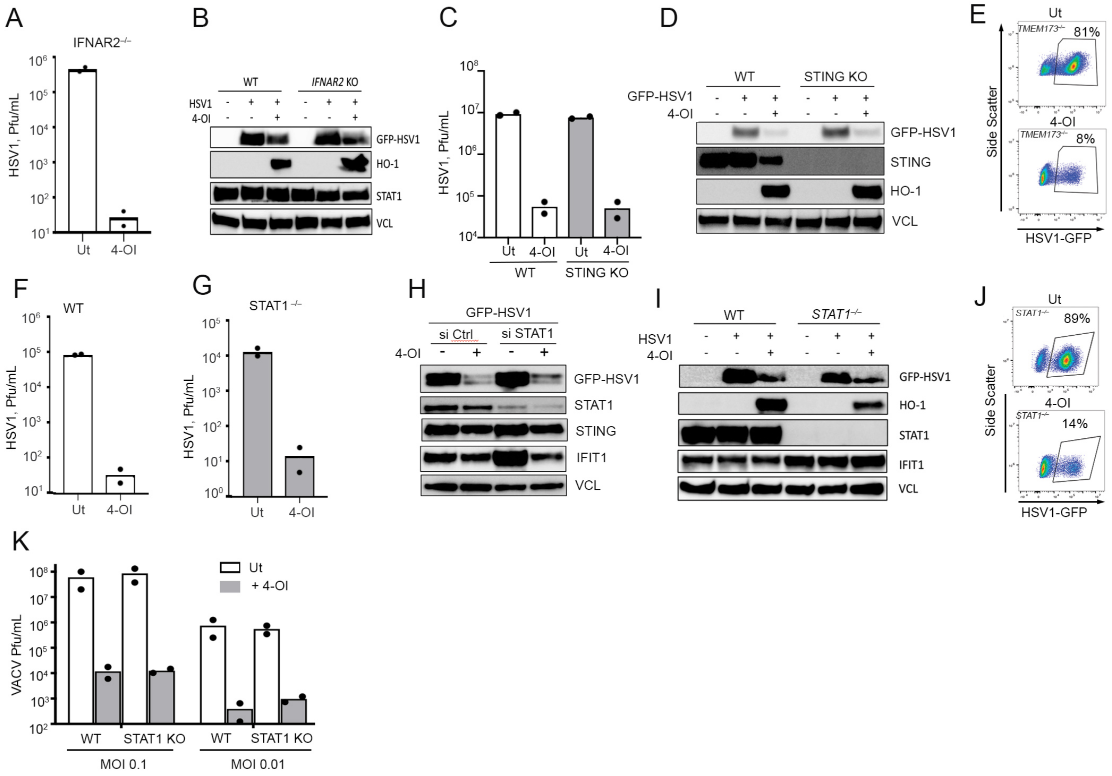

Supplementary Figure 6. 4-OI induces an anti-viral program independently of IFNs but dependent on NRF2. **A-K)** WT HaCaT cells or HaCaT cells deficient in IFNAR2, STING or STAT1 were either treated with DMSO alone (Ut) or with 4-OI at 125µM before being infected with GFP-expressing HSV1 (GFP-HSV1) at MOI 0.01. After 20 hours, supernatants were collected for analysis by plaque assay (A, C, F, and G). In parallell experiment, cells were either lysed for immunoblotting (B, D, H and I) using indicated antibodies or analyzed by flow cytometry (E and J). **K)** Cells were treated with 4-OI at 150µM before infection with VACV WR strain at MOI 0.01 and 0.1. After 20 hours, supernatants were collected and analyzed by plaque assay. Data in A-K display one experiment representative of two independent experiments each performed in dublicates with bars indicating mean.

Supplementary Figure 7.

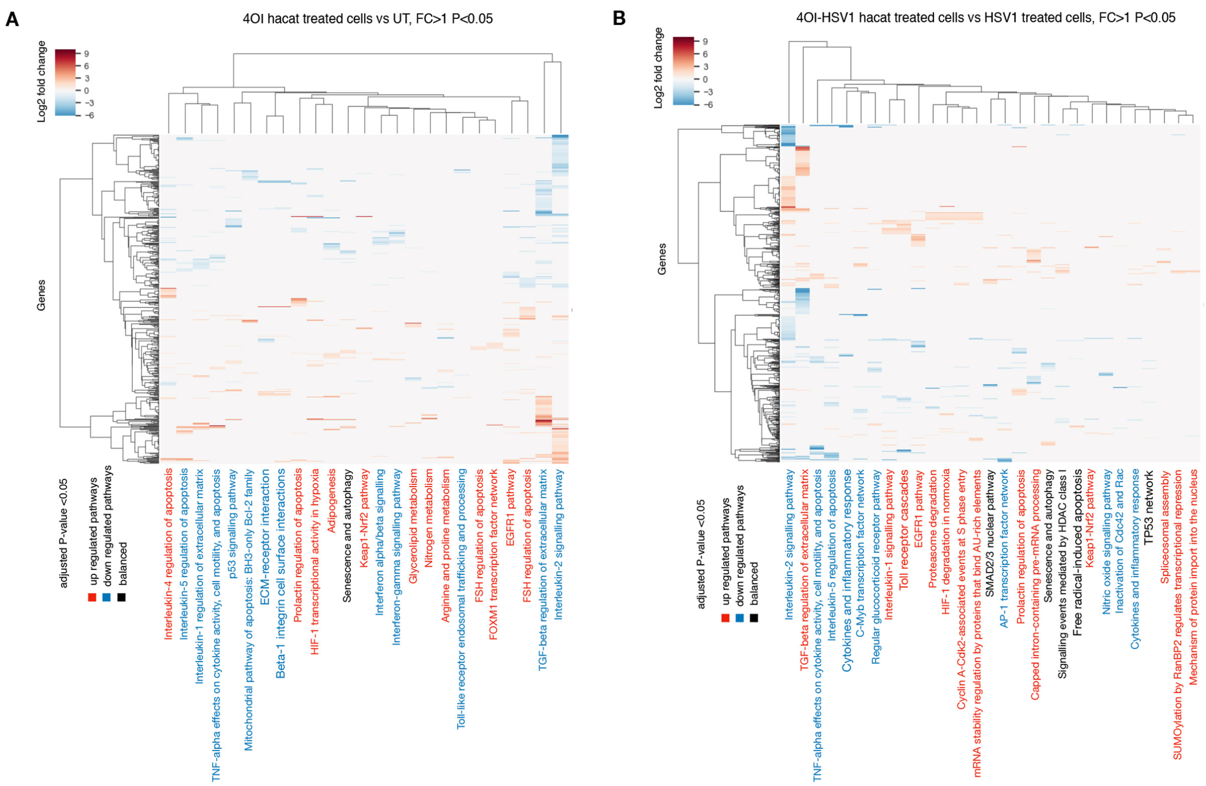

Supplementary Figure 7. Transcriptome pathway analysis of 4-OI treated HaCaT cells
Disregulated pathways significant at adjusted P-value<0.05 in HACAT cells after 4OI treatment relative to un-treated (UT) cells (A) and in HACAT cells after 4OI and HSV1 exposure relative to HSV1 infected cells (B). Both heat maps indicate the presence of a gene involved in each pathway by the genes colour coded expression value. Genes are filtered for |log 2(FoldChange)|>1 and adjusted p-value<0.05. Names of pathways with a higher number of up-regulated genes are shown in red, those with a higher number of down-regulated genes in blue,and are written in black if the number of up- and down-regulated genes are equal.

Supplementary Figure 8.

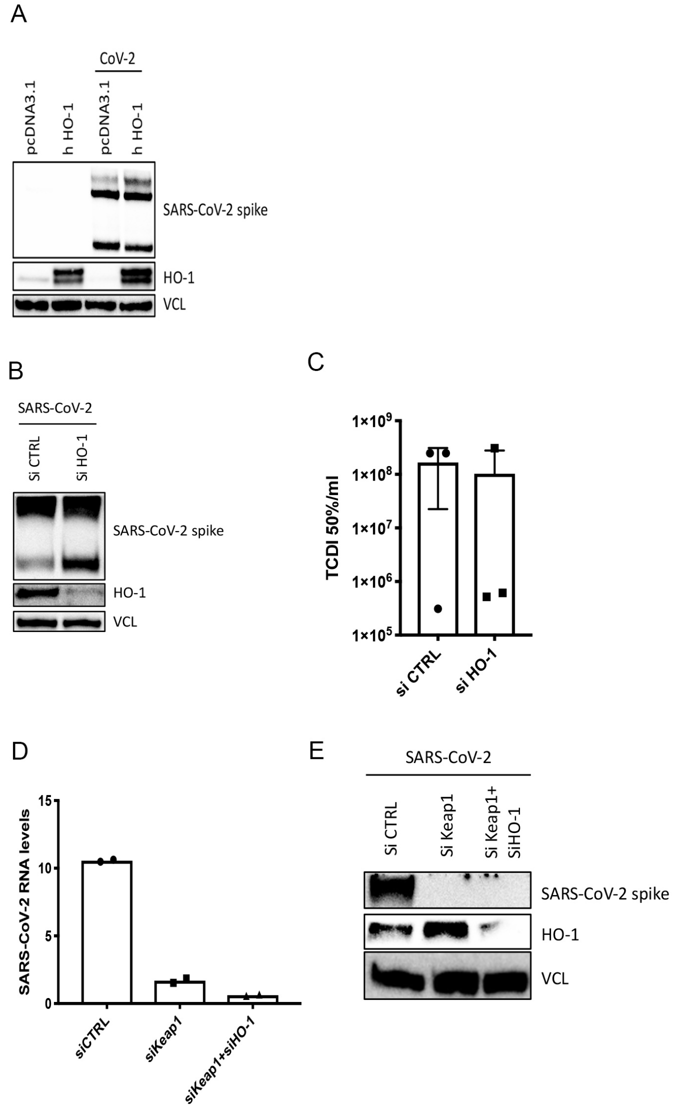

Supplementary Figure 8. HO-1 is not an important restriction factor for SARS-CoV2.
A) Vero cells were transfected with either an empty expression vector or one that encoded expression of human HO-1. Cells were then infected with SARS-CoV2 for 48 hours before analysis by immunoblotting. Data are one representative of two independent experiments.
B+C) Calu-3 cells were treated with ctrl siRNA or siRNA targeting HO-1. Cells were then infected with SARS-CoV2 for 48 hours before analysis of cell pellet by immunoblotting and of cell supernatants by TCID50 analysis.Data are one representative of two independent experiments.
D+E) Calu-3 cells were treated with indicated siRNAs before infection with SARS-CoV2 for 48 hours. Cell pellets were then analysed by qPCR or by immunoblotting.Data are one representative of two independent experiments.
All bars indicate mean and all error bars indicate s.e.m.

Supplementary Figure 9.

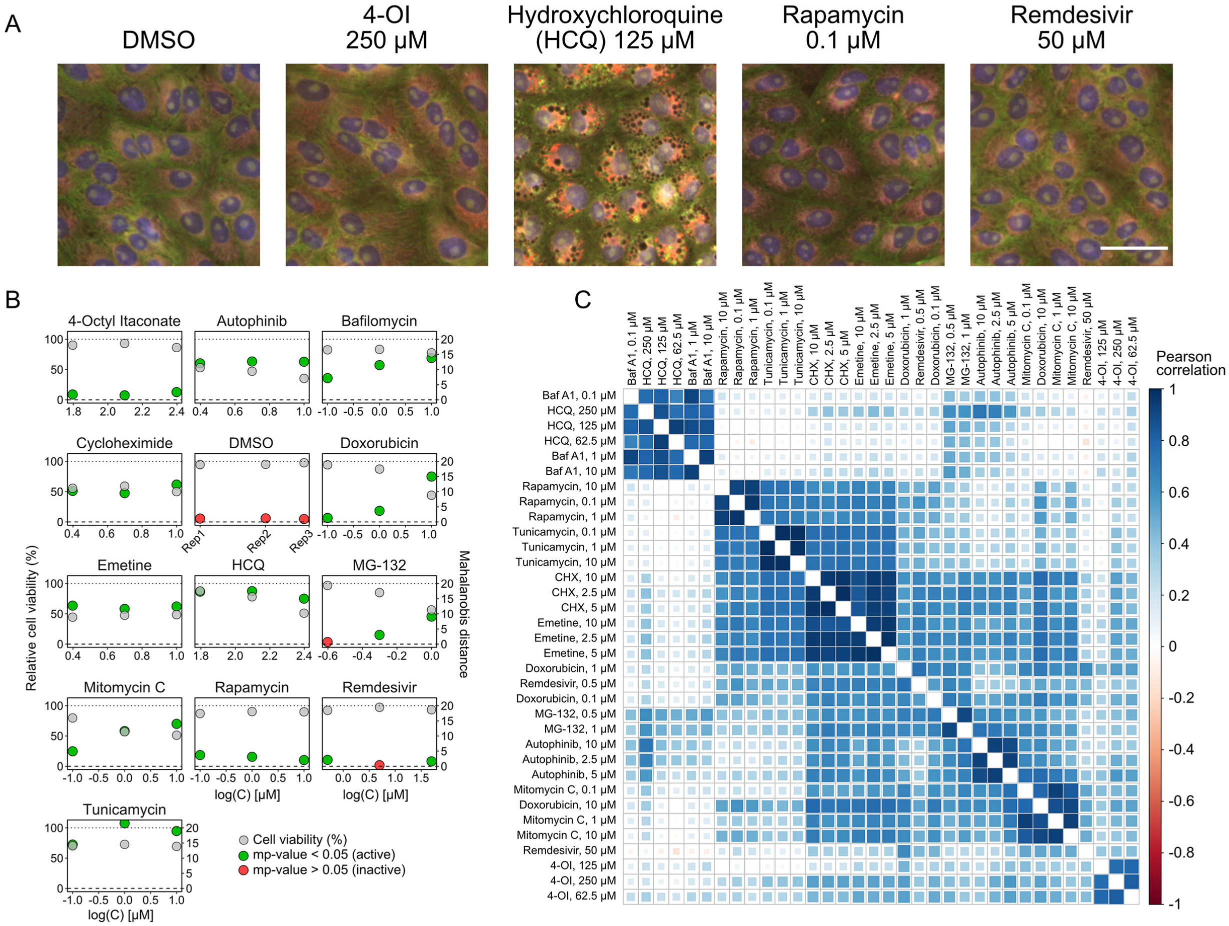

Supplementary Figure 9. Morphological profiling of 4-OI in Vero cells.
Vero E6 cells were subjected to morphological profiling. Compounds were dosed at three concentrations, either 2-fold or 10-fold dilutions in quadruplicates for 24 hours before staining with MitoTracker Deep Red for 30 minutes. The cells were then fixed and permeabilized before staining with the multiplex staining solution and imaged in 5 channels. Image analysis in Cell Profiler 2.1.1 extracted 1476 features related to cell morphology, which constitutes the morphological profile.
A) Representative images from the profiling experiment. Scale bar = 50 μM.
B) Cell viability and profile activity measured as the Mahalanobis distance to DMSO negative controls. The mp-value (see method section) is used to determine significant activity. 4-OI has low but significant activity (mp-value < 0.05) without loss of cell viability. DMSO is quadruplicates of wells kept separate from the DMSO wells used for normalization.
C) Correlation matrix of Pearson correlations clustered by hierarchical clustering with average linkage for active profiles (mp-value < 0.05). The three doses of 4-OI clusters together but show no large correlations to other modulators of various cell processes such as translation (cycloheximide (CHX), emetine), transcription (doxorubicin, mitomycin c) or lysosomal acidification (bafilomycin A1 (Baf A1), hydroxychloroquine (HCQ)).

Supplementary Figure 10.

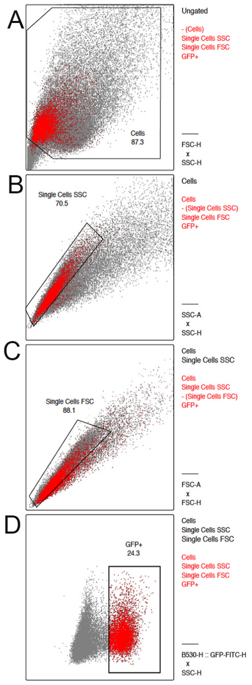

Supplementary Figure 10. Gating strategy for flowcytometric assesment of viral infection using GFP-expressing virus strains. A) Ungated population. B) Gating single cells using SSC. C) Gating single cells using FSC. D) Final population with GFP positive and negative cells.

Supplementary Figure 11
Uncropped WBs

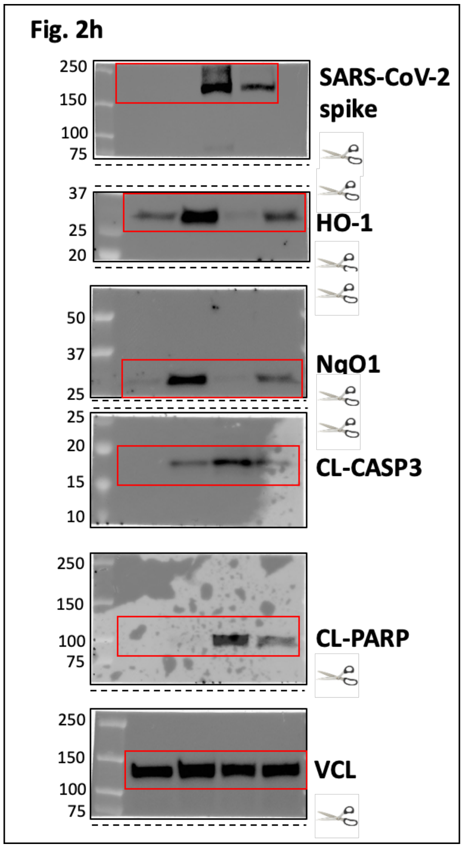

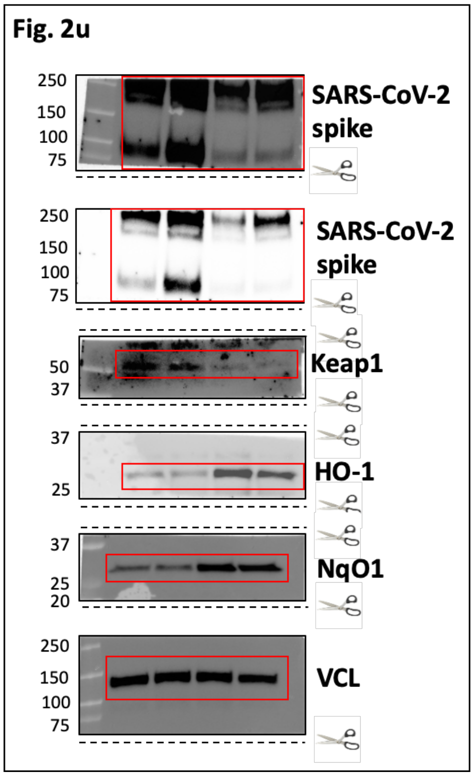

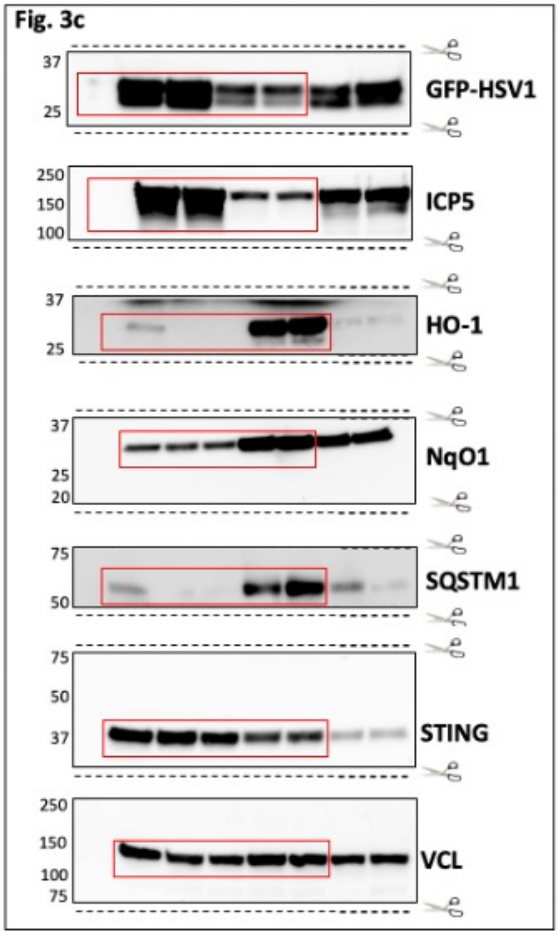

Fig. 3c
37
25
GFP-HSV1
250
150
100
ICP5
37
25
HO-1
37
25
20
NqO1
75
50
SQSTM1
75
50
37
STING
250
150
100
75
VCL

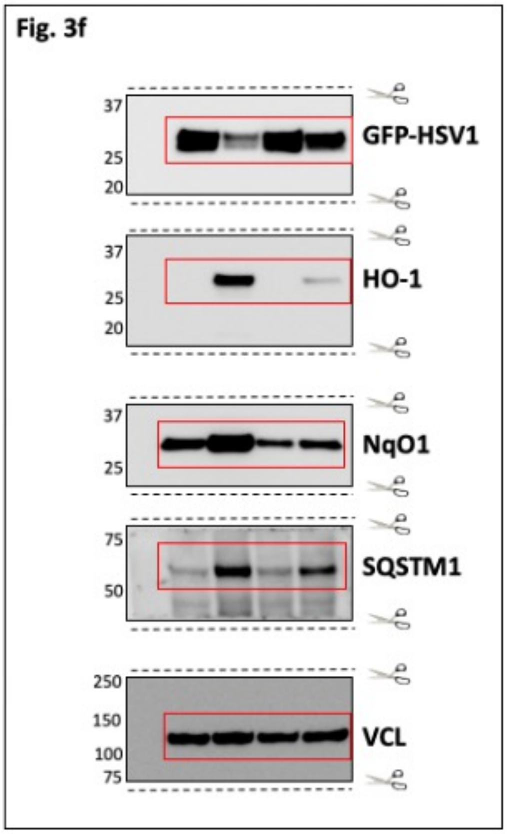

Fig. 3f
37
25
20
GFP-HSV1
37
25
20
HO-1
37
25
NqO1
75
50
SQSTM1
250
150
100
75
VCL

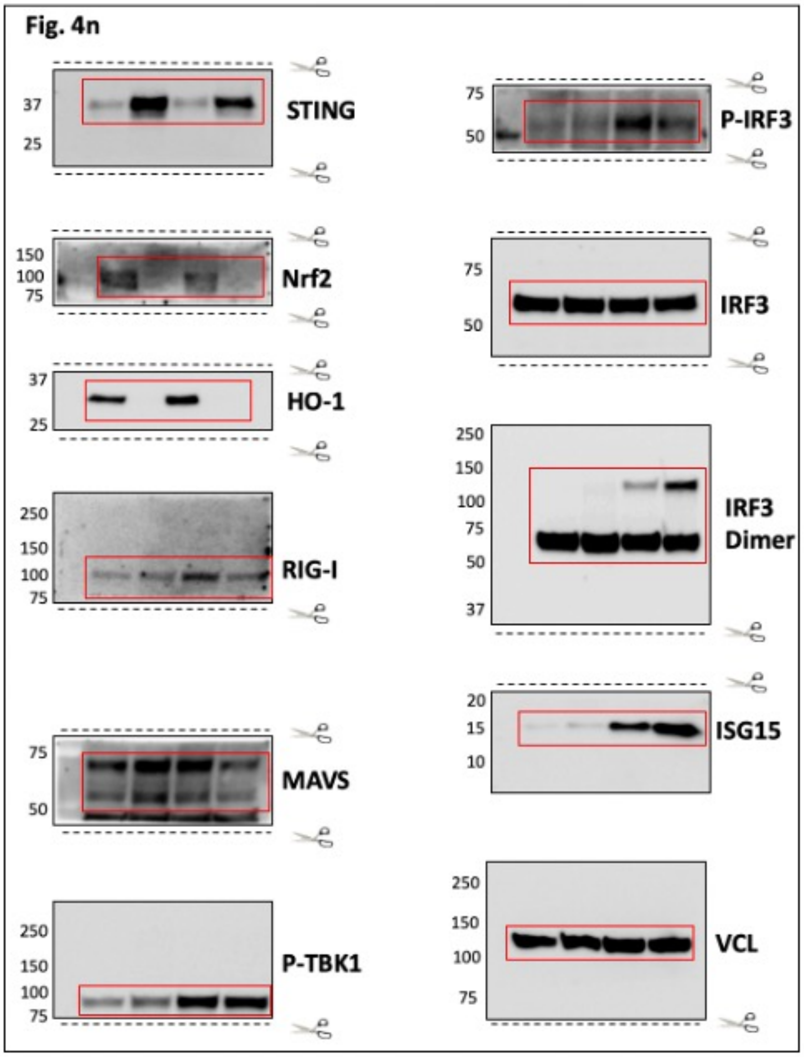

Fig. 4n
37
25
STING
75
50
P-IRF3
150
100
75
Nrf2
75
50
IRF3
37
25
HO-1
250
150
100
75
IRF3
Dimer
50
37
250
150
100
75
RIG-I
20
15
10
ISG15
75
50
MAVS
250
150
100
75
VCL
250
150
100
75
P-TBK1

Fig. 4o

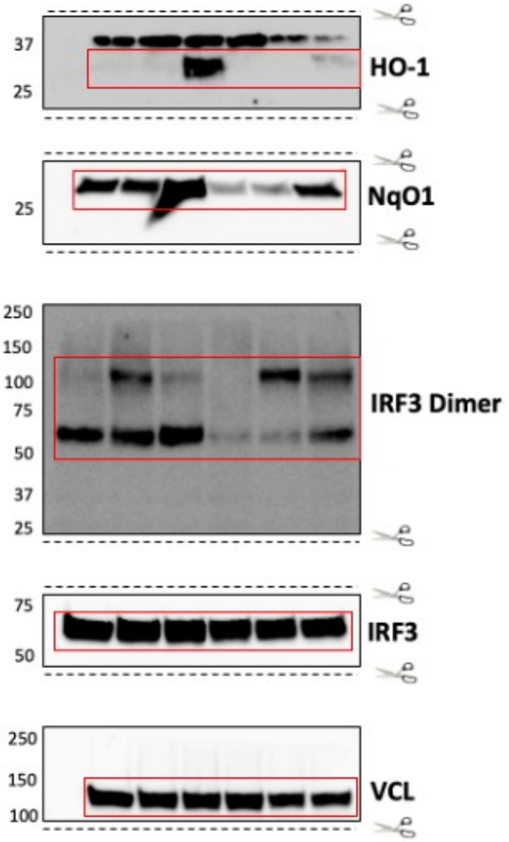

Fig. 4q

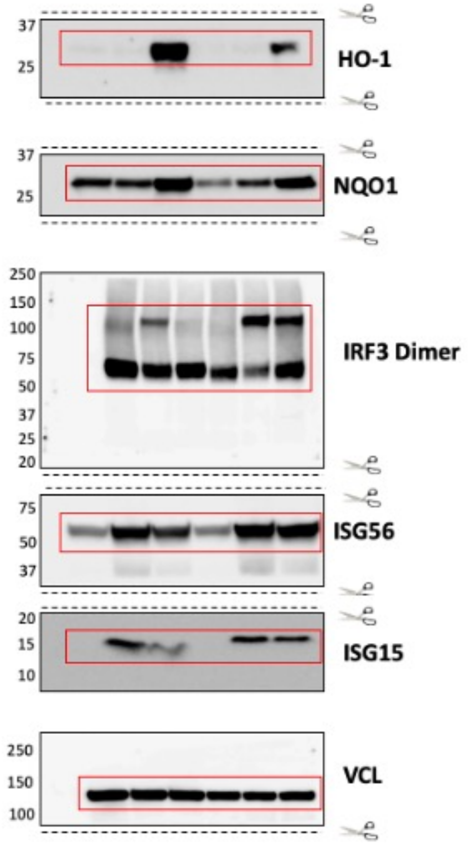

Fig. 4t

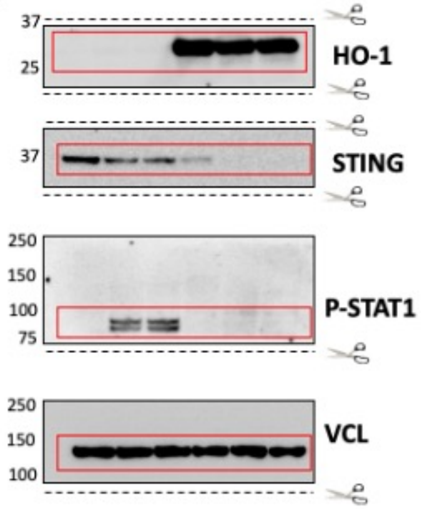

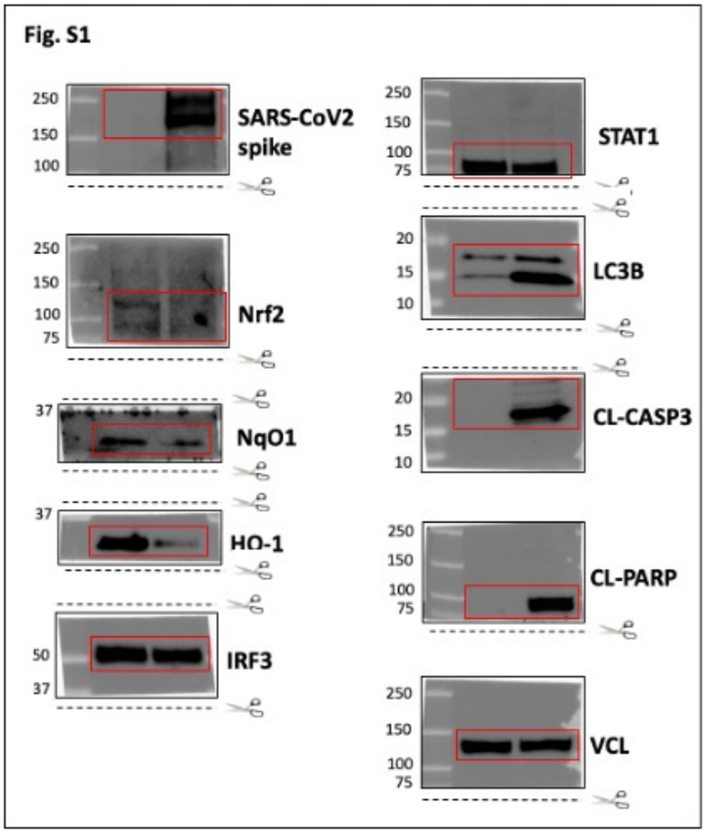
Fig. S1
250
150
100
SARS-CoV2
spike
250
150
100
75
Nrf2
37
NqO1
37
HO-1
50
37
IRF3
250
150
100
75
STAT1
20
15
10
LC3B
20
15
10
CL-CASP3
250
150
100
75
CL-PARP
250
150
100
75
VCL

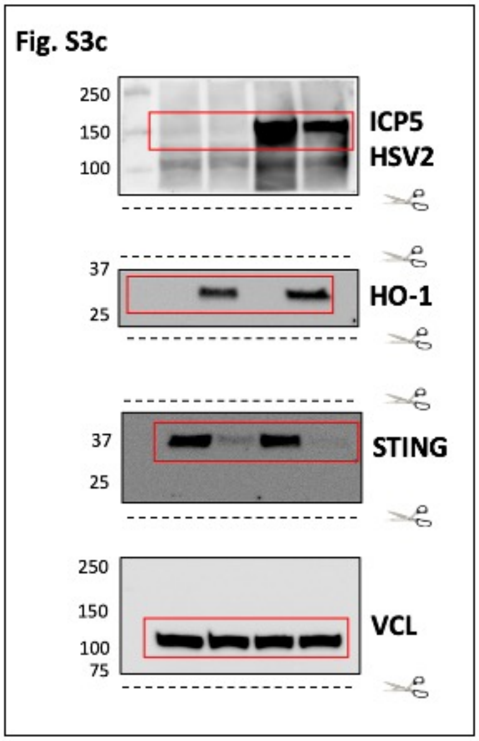
Fig. S3c
250
150
100
ICP5
HSV2
37
25
HO-1
37
25
STING
250
150
100
75
VCL

Fig. S6b

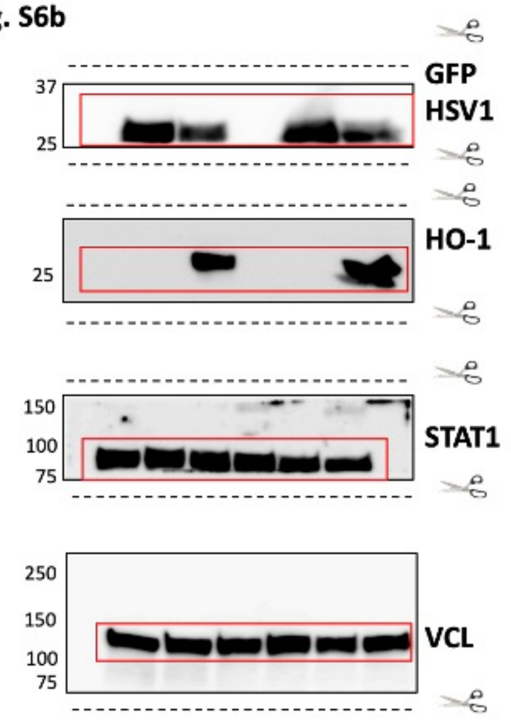

Fig. S6d

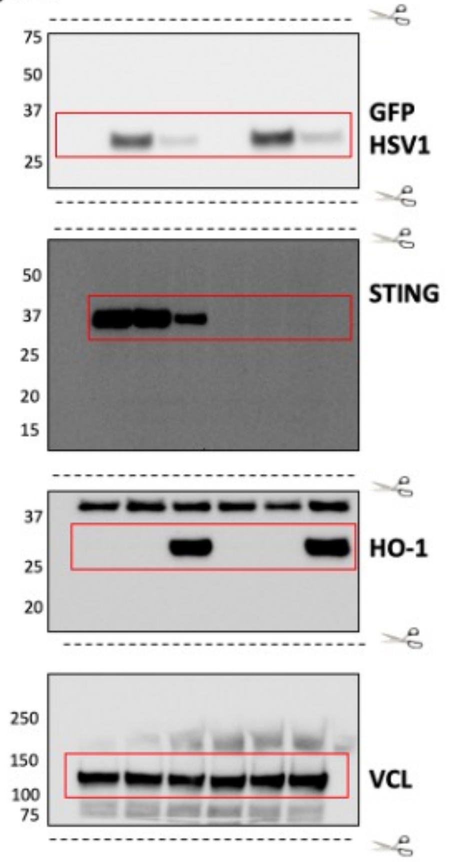

Fig. S6h

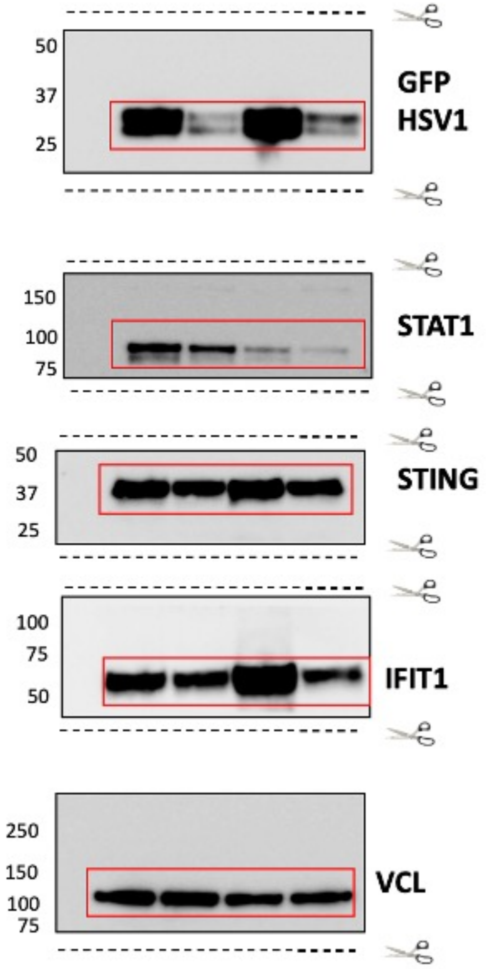

Fig. S6i

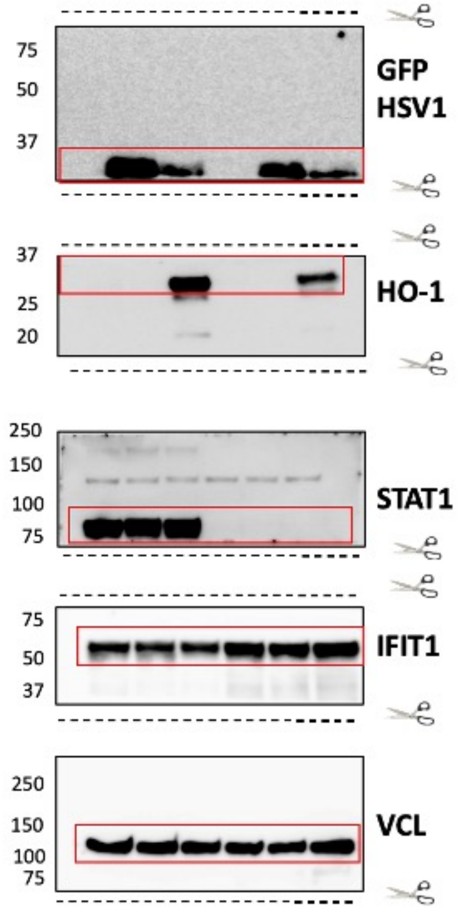

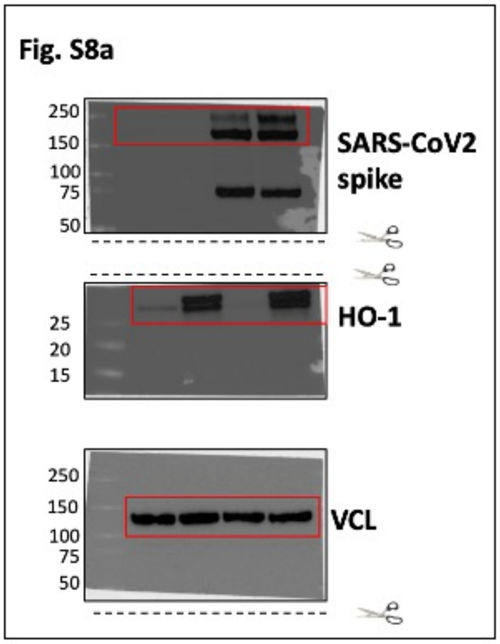
Fig. S8a
250
150
100
75
50
SARS-CoV2 spike
25
20
15
HO-1
250
150
100
75
50
VCL

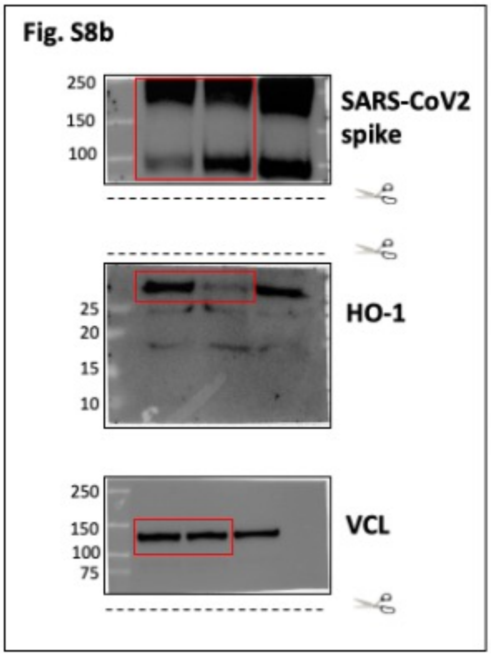
Fig. S8b
250
150
100
SARS-CoV2 spike
25
20
15
10
HO-1
250
150
100
75
VCL

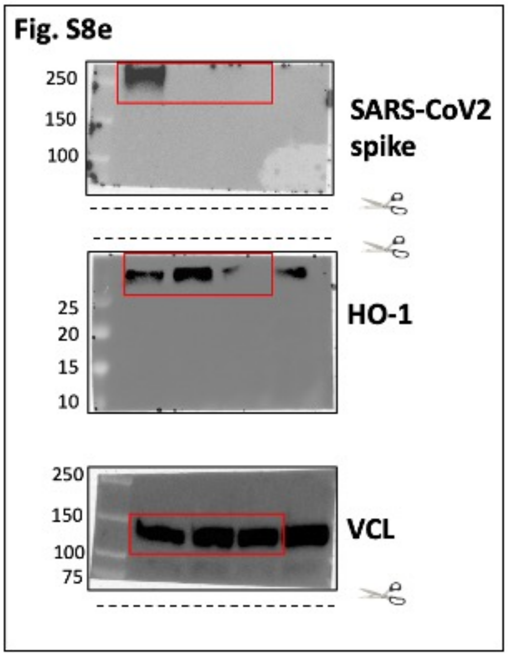
Fig. S8e
250
150
100
SARS-CoV2 spike
25
20
15
10
HO-1
250
150
100
75
VCL
