# Supplementary material for: SARS-CoV2-mediated suppression of NRF2-signaling reveals potent antiviral and anti-inflammatory activity of 4-octyl-itaconate and dimethyl fumarate
Source: Nat Commun. 2020 Oct 2;11:4938. doi: 10.1038/s41467-020-18764-3 (PMC7532469; doi:10.1038/s41467-020-18764-3)
Supplement: Supplementary file 3 — Reporting Summary [file 41467_2020_18764_MOESM3_ESM.pdf]

nature research

Corresponding author(s): Christian Kanstrup Holm and David Olagnier

Last updated by author(s): Aug 25, 2020

Reporting Summary

Nature Research wishes to improve the reproducibility of the work that we publish. This form provides structure for consistency and transparency in reporting. For further information on Nature Research policies, see our Editorial Policies and the Editorial Policy Checklist.

Statistics

For all statistical analyses, confirm that the following items are present in the figure legend, table legend, main text, or Methods section.

n/a	Confirmed	
☐	☒	The exact sample size (*n*) for each experimental group/condition, given as a discrete number and unit of measurement
☐	☒	A statement on whether measurements were taken from distinct samples or whether the same sample was measured repeatedly
☐	☒	The statistical test(s) used AND whether they are one- or two-sided<br>*Only common tests should be described solely by name; describe more complex techniques in the Methods section.*
☒	☐	A description of all covariates tested
☒	☐	A description of any assumptions or corrections, such as tests of normality and adjustment for multiple comparisons
☐	☒	A full description of the statistical parameters including central tendency (e.g. means) or other basic estimates (e.g. regression coefficient) AND variation (e.g. standard deviation) or associated estimates of uncertainty (e.g. confidence intervals)
☐	☒	For null hypothesis testing, the test statistic (e.g. *F*, *t*, *r*) with confidence intervals, effect sizes, degrees of freedom and *P* value noted<br>*Give P values as exact values whenever suitable.*
☒	☐	For Bayesian analysis, information on the choice of priors and Markov chain Monte Carlo settings
☒	☐	For hierarchical and complex designs, identification of the appropriate level for tests and full reporting of outcomes
☐	☒	Estimates of effect sizes (e.g. Cohen's *d*, Pearson's *r*), indicating how they were calculated

Our web collection on statistics for biologists contains articles on many of the points above.

Software and code

Policy information about availability of computer code

Data collection	No software used
Data analysis	About that section you need to fill in:<br>1. RNA-seq data was obtained from Blanco-Melo (doi: https://doi.org/10.1101/2020.03.24.004655) (ref 14)<br>2. Re-analysis of RNA-seq data: raw read-counts differential expression values were calculated using DESeq2 (Ref 38)<br>3. For Heat map in figure 1, we used Python3.7 and seaborn cluster-map tools .<br>4. pathway enrichment analysis was done using Enrichr (Ref 39)<br>5. For cloud analysis, we used the STRING database(Ref 40)<br>6. For network visulization we used Cytoscape (Ref 41) version 3.7.

For manuscripts utilizing custom algorithms or software that are central to the research but not yet described in published literature, software must be made available to editors and reviewers. We strongly encourage code deposition in a community repository (e.g. GitHub). See the Nature Research guidelines for submitting code & software for further information.

Data

Policy information about availability of data

All manuscripts must include a data availability statement. This statement should provide the following information, where applicable:

- Accession codes, unique identifiers, or web links for publicly available datasets
- A list of figures that have associated raw data
- A description of any restrictions on data availability

For COVID19 data set analysis (Fig. 1) the RNA-seq data was obtained from an already available dataset from Blanco-Melo et al,. /([https://doi.org/10.1101/2020.03.24.004655])(14). Transcriptome analysis of lung autopsies obtained from five individual COVID-19 patients (Desai et al., 2020)(15) [https://

doi.org/10.1101/2020.07.30.20165241] GEO accession code GSE150316. For RNA sequencing, files generated for analysis of HaCaT cells infected with HSV in the presence or absence of 4-OI have been deposited to the data depository database (CNGBdb, [https://db.cngb.org]) with the following accession number : CNP0001039. Source data are provided with this paper.

Field-specific reporting

Please select the one below that is the best fit for your research. If you are not sure, read the appropriate sections before making your selection.

[x] Life sciences [ ] Behavioural & social sciences [ ] Ecological, evolutionary & environmental sciences

For a reference copy of the document with all sections, see nature.com/documents/nr-reporting-summary-flat.pdf

Life sciences study design

All studies must disclose on these points even when the disclosure is negative.

Sample size	No sample size calculations were performed. Sample sizes were chosen based on previous experience with in vitro cell cultures.
Data exclusions	No data were excluded
Replication	All experiments were replicated at least once in an independent setup. All attempts to replicate experiments were successful.
Randomization	All experiments were performed in vitro using unbiased measuring techniques (qPCR etc.). Therefore, randomization was not used.
Blinding	All experiments were performed in vitro using unbiased measuring techniques that are independent of the scientist performing the experiment (qPCR etc.). Therefore, blinding was not used.

Reporting for specific materials, systems and methods

We require information from authors about some types of materials, experimental systems and methods used in many studies. Here, indicate whether each material, system or method listed is relevant to your study. If you are not sure if a list item applies to your research, read the appropriate section before selecting a response.

Materials & experimental systems

n/a	Involved in the study	
[ ]	[x]	Antibodies
[ ]	[x]	Eukaryotic cell lines
[x]	[ ]	Palaeontology and archaeology
[x]	[ ]	Animals and other organisms
[ ]	[x]	Human research participants
[x]	[ ]	Clinical data
[x]	[ ]	Dual use research of concern

Methods

n/a	Involved in the study	
[x]	[ ]	ChIP-seq
[ ]	[x]	Flow cytometry
[x]	[ ]	MRI-based neuroimaging

Antibodies

Antibodies used	anti-Nrf2 (12721, Cell Signaling 1:1000), anti-TBK1/NAK (3013, Cell Signaling 1:1000), anti-phospho-TBK1/NAK (5483, Cell Signaling 1:1000), anti-SQSTM1/p62 (8025, Cell Signaling 1:1000), anti-IRF3 (11904, Cell Signaling 1:1000), anti-phospho-IRF3 (4947, Cell Signaling 1:500), anti-HO-1 (5853, Cell Signaling 1:1000), anti-IFIT1 (14769, Cell Signaling 1:1000), anti-STING (13647, Cell Signaling 1:1000), anti-NqO1 (3187, Cell Signaling 1:1000), anti-STAT1 (9172, Cell signaling, 1:1000), SARS-CoV2 spike anti-body (GeneTex, cat# GTX632604, 1:1000), and anti-Vinculin (18799, Cell Signaling 1:1000) used as loading control. After three washes in PBST, secondary antibodies, peroxidase-conjugated F(ab)2 donkey anti-mouse IgG (H+L) (1:10000) or peroxidase-conjugated F(ab)2 donkey anti-rabbit IgG (H+L) (1:10000) (Jackson ImmunoResearch)
Validation	anti-Nrf2 (12721, Cell Signaling 1:1000): Validated by molecular size and by siRNA targeting NRF2 (Fig. 4n)<br>anti-TBK1/NAK (3013, Cell Signaling 1:1000): Validated by molecular size.<br>anti-phospho-TBK1/NAK (5483, Cell Signaling 1:1000): Validated by molecular size.<br>anti-SQSTM1/p62 (8025, Cell Signaling 1:1000): Validated by molecular size.<br>anti-IRF3 (11904, Cell Signaling 1:1000): Validated by molecular size.<br>anti-phospho-IRF3 (4947, Cell Signaling 1:500): Validated by molecular size.<br>anti-HO-1 (5853, Cell Signaling 1:1000), : Validated by molecular size and siRNA mediated knockdown (Suppl. Fig. 8B)<br>anti-IFIT1 (14769, Cell Signaling 1:1000): Validated by molecular size.<br>anti-STING (13647, Cell Signaling 1:1000): Validated by molecular size and Crispr KO (Suppl. Fig. 6D)<br>anti-NqO1 (3187, Cell Signaling 1:1000): Validated by molecular size.<br>anti-STAT1 (9172, Cell signaling, 1:1000): Validated by molecular size and Crispr KO and siRNA Knockdown(Suppl. Fig. 6H+I)<br>anti-SARS-CoV2 spike anti-body (GeneTex, cat# GTX632604, 1:1000): Validated by molecular size and with and without SARS-CoV2

	infection. (Fig. 2h and supplementary Figure 1)<br>anti-Vinculin (18799, Cell Signaling 1:1000): Validated by molecular size.

Eukaryotic cell lines

Policy information about cell lines

Cell line source(s)	HaCaT (ATCC), NuLi-1 cells (ATCC), Calu-3 cells(ATCC), Vero Cells (ATCC),4T1 cells (ATCC), 786-O cells(ATCC), HEK293(ATCC), A549 (ATCC), Huh-7 (ATCC).
Authentication	No authentication
Mycoplasma contamination	All cell lines were tested routinely for Mycoplasma infection. All tests were negative.
Commonly misidentified lines<br>(See ICLAC register)	No commonly misidentified cell lines were used in this study.

Human research participants

Policy information about studies involving human research participants

Population characteristics	All patients were positive for SARS-CoV2 by PCR from throat swab and admitted to the ICU and receiving ventilatory support due to severe pneumonia with a component of acute respiratory distress syndrome (ARDS). P1 27-years old male, day 14 at in ICU. P2 57-year-old female, day 9 in ICU. P3 43 years-old male, day 5 in ICU. P4 28 years-old female, day 3 in ICU.
Recruitment	The healthy control PBMCs were achieved from anonymous donors from the Blood Bank at Aarhus University Hospital. We have used these PBMCs for many previous studies and experiments (references: Andersen et al J. Exp.Med 2015; Ogunjimi et al J. Clin Invest 2017) and in our experience they are representative concerning age and sex distribution and immune responses. We don't think there is any major bias present, if anything that donors were healthy since they were blood donors but indeed the patients were also selected based on criteria of being previously healthy without major chronic diseases, malignancy or immunosuppressive treatments.<br><br>Patients were recruited per protocol (approved by The Danish National Committee in Health Research Ethics (1-10-72-80-20)) from a central University Hospital in Denmark based on the criteria that they were diagnosed with COVID-19 by positive PCR for SARS-CoV-2 from throat swab, were admitted to the ICU with oxygen requirement and ventilatory support and had a degree of acute respiratory distress syndrome (ARDS) with abnormalities on x-ray. Furthermore they were all < 60 years of age, previously healthy and without known co-morbidities for severe COVID-19 and also without known malignant disease and in no immunosuppressive treatment prior to or at the time of COVID-19 Patients or their family members were asked to participate and given written and oral information before providing written informed consent according to the Helsinki Declaration criteria.<br>We don't believe there was any self selection bias or other major biases involved in the selection process.
Ethics oversight	Danish National Committee in Health Research Ethics (1-10-72-80-20) and the Danish Data protection Agency in accordance with the ethical standards of the Helsinki Declaration. Written informed consent was obtained from all study participants.

Note that full information on the approval of the study protocol must also be provided in the manuscript.

Flow Cytometry

Plots

Confirm that:

- [x] The axis labels state the marker and fluorochrome used (e.g. CD4-FITC).
- [x] The axis scales are clearly visible. Include numbers along axes only for bottom left plot of group (a 'group' is an analysis of identical markers).
- [x] All plots are contour plots with outliers or pseudocolor plots.
- [x] A numerical value for number of cells or percentage (with statistics) is provided.

Methodology

Sample preparation	Briefly, cells were harvested, washed with FACS buffer (PBS, 0.01% sodium azide, and 0.1% BSA) and fixed with paraformaldehyde 4% in PBS for 10 min. After extensive washing with FACS buffer, 2x104 cells were scored and analyzed in a FACSCalibur flow cytometer (BD Sciences) per experimental condition in triplicates
Instrument	FACSCalibur flow cytometer (BD Sciences)
Software	FlowJo

Cell population abundance	Not relevant
Gating strategy	We will provide a supplementary figure on gating strategy in the revised manuscript

☒ Tick this box to confirm that a figure exemplifying the gating strategy is provided in the Supplementary Information.
